# Sulfidation mechanism of ZnO roasted with pyrite

**DOI:** 10.1038/s41598-018-27968-z

**Published:** 2018-06-22

**Authors:** Wei Liu, Lin Zhu, Junwei Han, Fen Jiao, Wenqing Qin

**Affiliations:** School of Minerals Processing and Bioengineering, Central South University, 410083 Changsha, Hunan China

## Abstract

Sulfidation is a widely used technology to improve the floatability of oxidized metal minerals or to stabilize the heavy metals in various wastes. The sulfidation mechanism of ZnO with pyrite was detailedly studied by thermodynamic calculation and roasting experiments. The sulfidation behaviors, phase transformations, microscopic morphology and surface properties were investigated by TG-DSC, ICP, XRD, SEM-EDS, and XPS analysis. The results indicate that the nature of the sulfidation is the reaction of ZnO with the gaseous sulfur generated by the decomposition of pyrite. Pyrite instead of sulfur as the sulfidizing agent can not only relieve the volatilization loss of sulfur but also enhance the formation of liquid phase and thus facilitate the growth of ZnS particles. The sulfidation reaction belongs to surface chemical reaction and relates to the migration of oxygen from the inside of ZnO to its surfaces. The presence of carbon not only eliminates the release of SO_2_, but also decreases the decomposition temperature of pyrite and promotes the sulfidation of ZnO. The addition of Na_2_CO_3_ promotes the sulfidation of ZnO at lower temperatures (below 850 °C) and enhances the growth of ZnS particles but has a negative effect on the sulfidation at higher temperatures.

## Introduction

Zinc is an important base metal required for various applications and is mainly recovered from primary sulfide ores *via* flotation and metallurgical processes^1^. With the rapid development of nonferrous industry, high-grade lead-zinc sulfide ores are exhausted day by day, and correspondingly, millions of tons of heavy metal containing wastes are generated annually from nonferrous smelting process in the world, such as zinc leaching residue, zinc ash, and lead smelting slag^2,3^. In general, these smelting wastes contain plenty of valuable metals whose content are more than that of primary ores and thus are considered as important secondary resources for relieving the global metal supply. Furthermore, the huge quantities of wastes can potentially impose a negative impact to the environment because of the possibility of releasing heavy metal elements, such as Pb, Cd and As, based on that fact that metals in smelting wastes are rarely in sulfides but are in oxides and oxidized compounds, which are more soluble than their sulfide counterparts^4,5^. Therefore, the concern for stockpiling the wastes is not only the loss of valuable metals but also environmental threats. To solve the problems of resource shortage and environmental pollution, a series of hydrometallurgical, pyrometallurgical, and their combined routes have been developed for extracting metals from heavy metal containing wastes^6–12^. Unfortunately, these technologies have not been widely applied for mass production due to the presence of some technical, economical, and environmental drawbacks. For instance, pyrometallurgical methods are energy intensive and require a complicated dust collection system, while hydrometallurgical processes usually result in considerable amounts of wastewater and residues containing various heavy metals.

As is well-known, flotation is an economical green technology and is widely used for metal recovery from primary sulfide ores. Since the surfaces of oxidized minerals are generally more hydrophilic than that of corresponding sulfide minerals, it is difficult to directly recover metals from smelting wastes and oxide ores by flotation^13^. If an effective and reliable technology to convert the metal oxides and oxidized compounds into sulfides is developed, the metals can be successfully recovered by conventional flotation. For this purpose, sulfidation has received much attention as a possible generic technology for heavy metal recovery. At present, a number of sulfidation technologies, including surface sulfidation with Na_2_S^14–16^, mechanochemical sulfidation^17–19^, hydrothermal sulfidation^20–22^, and sulfidation roasting^23–25^, have been developed for improving the floatability of metal oxides. The most common approach commercially is to sulfidize the surfaces of oxide minerals with Na_2_S prior to flotation, after which the hydrophilicity of their surfaces is significantly decreased due to the presence of chemisorbed sulfide ions^26^. A fatal drawback of this process is that the dosage of sulfidizing agent is highly dependent on the time of conditioning, procedures of mixing, and other variables, resulting in poor reproducibility in a plant situation. An excess sulfidizing agent functions as a depressant for oxidized minerals because the adsorption of divalent sulfide ions on their surfaces increases the negative charge, which will prevent the adsorption of collector onto mineral surfaces^27^.

Mechanochemical and hydrothermal sulfidation may be viewed as green technologies for the recovery and stabilization of the heavy metals in wastes and are recently studied for the transformation of metal oxides and oxidized compounds to corresponding sulfides with sulfur. Flotation results indicated that the synthetic metal sulfides showed a poor flotation performance although a high sulfidation extent and low heavy metal dissolubility could be achieved after mechanochemical or hydrothermal sulfidation. This is because the zinc sulfide crystals formed by the two techniques have fine particle size and poor crystalline structure^28–30^. By contrast, sulfidation roasting is a more commercially feasible process to obtain metal sulfides with sufficient particle size and good crystalline structure^31^, because high temperature favors the formation and growth of mineral crystals, and hence shows better results for the metal recovery from low-grade oxide ores or smelting wastes by flotation. For example, Li *et al*.^32^ investigated the recovery of lead and zinc from low-grade lead-zinc oxide ore by sulfidation roasting and flotation process. The results showed 79.5% Pb and 88.2% Zn were recovered by this process, while the concentrate contained 10.2% Pb and 38.9% Zn. Zheng *et al*.^33^ employed sulfidation roasting and flotation process to recycle valuable metals from zinc leaching residues. The experimental results showed that a flotation concentrate with 39.13% Zn, 6.93% Pb and 973.54 g/t Ag was obtained, and the recovery rates of Zn, Pb and Ag were 48.38%, 68.23% and 77.41%, respectively.

Compared with oxide ores or residues, it is more difficult to recover valuable metals from smelting slags by sulfidation and flotation process, because it contains complex amorphous phases, resulting in the metal sulfides generated with low crystallinity and fine grains^34^. To enhance the floatability of generated metal sulfides, some researchers attempted to use a higher temperature roasting for accelerating the sulfidation reaction and particle growth during the sulfidation process. Harris *et al*.^35,36^ studied the sulfidation of nickeliferous lateritic ore with sulfur. They found that the Fe-Ni-S phase formed at lower temperatures was submicron in nature and heating to temperature between 1050 and 1100 °C not only allowed for the growth of the particles, but also facilitated the further reaction of iron sulfides with nickel oxides to iron oxides and nickel sulfides. Han *et al*.^37,38^ carried out the selective sulfidation of lead smelter slag at high temperatures. The results indicated that although the selective transformation of lead smelter slag could be achieved and the zinc sulfides with coarse grains and good crystallinity were generated after sulfidation roasting at above 1000 °C, a qualified zinc sulfide concentrate has never been obtained. The reasons are complicated and from many aspects, which need to be resolved. However, most of studies on sulfidation roasting have been restricted to the investigation in process optimization. As a result, it is necessary to establish a system of theoretical knowledge for further developing the sulfidation roasting technology of metal oxides.

The mechanism on the sulfidation of ZnO with sulfur at high temperatures has been studied in our previous work^39^. Since sulfur is volatile and easy to escape at high temperatures, the sulfidation with sulfur should be first carried out at a lower temperature, which limits sulfidation roasting being applied to the direct treatment of hot smelting slags. Previous studies indicate that pyrite can be directly used as a sulfidizing agent at high temperatures^38,40^. Therefore, it is essential to reveal the mechanism on the sulfidation of ZnO with pyrite, which however has not been systematically studied up to now. This study focused on the sulfidation of ZnO roasted with pyrite at high temperatures. Meanwhile, the effect of sodium carbonate as an additive on the sulfidation of ZnO was also studied in detail, based on the phenomenon that Na_2_CO_3_ addition favored the sulfidation of zinc contained in lead smelting slag and the growth of ZnS particles found by our previous studies. The thermodynamic analysis, sulfidation behaviors, phase transformations, microscopic morphology changes, and surface properties of the sulfidized ZnO were detailedly investigated by HSC combined with FactSage, TG-DSC, ICP, XRD, SEM-EDS, and XPS.

## Experimental Section

### Materials

ZnO and Na_2_CO_3_ powders used in this study are of analytical grade and were supplied by Sinopharm Chemical Reagent Co., Ltd. in China. Na_2_CO_3_ was used as an additive for investigating the effect of sodium salt on the sulfidation of ZnO. Pyrite with 47.9 wt.% S and 47.0 wt.% Fe obtained by hand sorting was used as the sulfidizing agent. A carbon powder with 73.0 wt.% C was used as the reducing agent. All the samples used were ground and sieved to smaller than 74 μm in advance.

### Methods

In the present study, roasting experiments were carried out in an elevator furnace, schematic of which had been shown in our previous article^41^. For each test, 10 g of zinc oxide was thoroughly mixed with scheduled mass ratio of pyrite, carbon, and sodium carbonate (if needed) powders using a mortar and pestle. The prepared mixture was loaded in an alundum crucible with a volume of 50 mL and sealed with a cover followed by iron wire bundling. The alundum crucible with sample was then put into the furnace. After the air in the furnace was excluded by introducing nitrogen of 2 L/min, the mixture was heated at a rate of 40 °C/min to a pre-set temperature and held at the condition for 2 h. When the roasting finished, the sample was taken out after it had cooled to below 100 °C under the atmosphere of nitrogen, then weighed, ground, and analyzed by a selective leaching and ICP analysis for the contents of zinc in sulfides and total zinc. The sulfidation extent of zinc was calculated as the following formula.

1 $$\alpha =\frac{M}{{M}_{T}}\cdot 100 \% $$

Where α is sulfidation extent, %; M is the zinc content in ZnS, wt.%, M_T_ is the total zinc content in roasted samples, wt.%.

The zinc content was determined with inductively coupled plasma (ICP, IRIS Intrepid II XSP). The crystal phase compositions were analyzed by X-ray powder diffraction (XRD, Germany Bruker-axs D8 Advance). The morphological characteristics were detected by scanning electron microscopy (SEM, Quanta FEG250) coupled with energy dispersive spectroscopy (EDS, Genesis XM2). Thermogravimetry-differential thermal analysis (TG-DSC) was performed using a thermal analyzer (PerkinElmer STA 8000) in flowing N_2_ at a heating rate of 10 °C/min from 30 °C to 1200 °C. X-ray photoelectron spectroscopy (XPS) study was carried out with a Thermo Scientific ESCALAB 250Xi using an Al Kα X-ray source. Binding energy calibration was based on C 1 s at 284.8 eV. The background of the spectrum was obtained using the Shirley method. A nonlinear least-square curve-fitting program (Avantage software 5.52) was used to deconvolve the XPS data.

## Results and Discussion

### Thermodynamic analysis

The sulfidation reactions of ZnO with pyrite (FeS_2_) that probably occurred during the roasting process are represented as follows:

2 $$2ZnO+3Fe{S}_{2}=2ZnS+3FeS+S{O}_{2}(g)$$

3 $$2ZnO+12/11Fe{S}_{2}=2ZnS+6/11F{e}_{2}{O}_{3}+2/11S{O}_{2}(g)$$

4 $$2ZnO+9/8Fe{S}_{2}=2ZnS+3/8F{e}_{3}{O}_{4}+1/4S{O}_{2}(g)$$

5 $$2ZnO+6/5Fe{S}_{2}=2ZnS+6/5FeO+2/5S{O}_{2}(g)$$

6 $$2ZnO+8/7Fe{S}_{2}=2ZnS+2/7FeS{O}_{4}+6/7FeO$$

7 $$2ZnO+14/13Fe{S}_{2}=2ZnS+2/13FeS{O}_{4}+6/13F{e}_{2}{O}_{3}$$

8 $$2ZnO+11/10Fe{S}_{2}=2ZnS+1/5FeS{O}_{4}+3/10F{e}_{3}{O}_{4}$$

9 $$2ZnO+2FeS=2ZnS+2FeO$$

**Figure 1:**
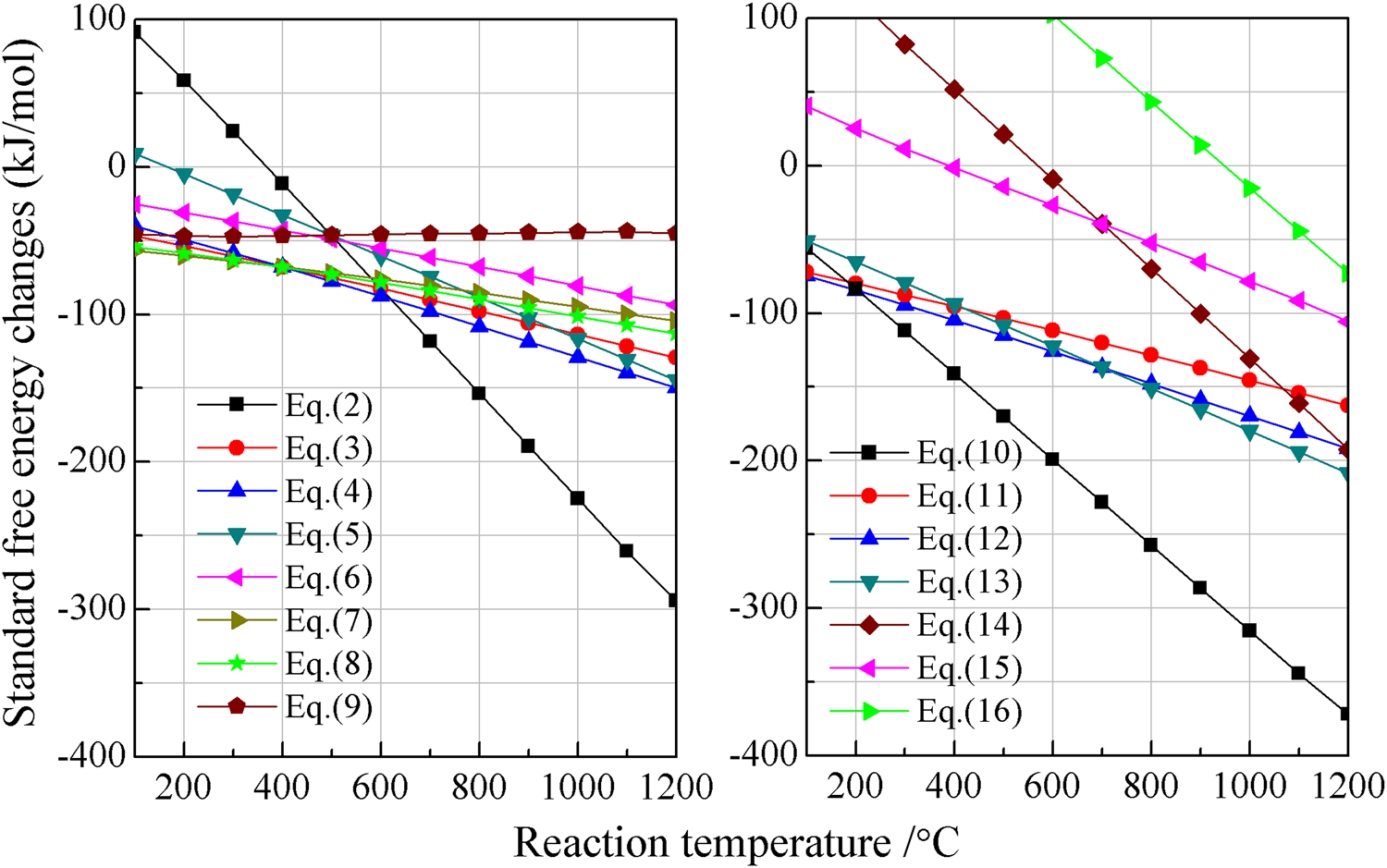
Standard free energy changes of the possible reactions as a function of temperature in the range of 100–1200 °C.

The standard free energy changes (ΔG) for the above reactions in the range of 100 °C to 1200 °C were calculated per 2 mole of ZnO by HSC Chemistry 5.0^42^. The results (Fig. 1) indicate that the sulfidation of ZnO with FeS_2_ is thermodynamically feasible in the temperature range investigated. With respect to the transformation of iron phases, Eqs. 7 and 8 exhibit relatively large negative ΔG at the range of 100 °C to 400 °C, with the values becoming more negative with increasing temperature and therefore the transformation of FeS_2_ to FeSO_4_ and Fe_2_O_3_/Fe_3_O_4_ is favourable. With further increasing temperature, the ΔG for Eq. 4 becomes more negative than Eqs. 7 and 8. When the temperature is above 600 °C, FeS will dominate in iron phases. In fact, most of sulfidation roasting processes were carried out in the range of 600 °C to1200 °C considering both kinetics and energy consumption. Therefore, ZnS, FeS, and SO_2_ are preferentially produced when ZnO roasted with stoichiometric FeS_2_. However, if the amount of FeS_2_ is insufficient, the FeS generated can further react with ZnO to form ZnS plus FeO.

The possible reactions of ZnO roasted with pyrite in the presence of carbon are listed as follows:

10 $$2ZnO+2Fe{S}_{2}+C=2ZnS+2FeS+C{O}_{2}(g)$$

11 $$2ZnO+Fe{S}_{2}+1/4C=2ZnS+1/2F{e}_{2}O{}_{3}+1/4C{O}_{2}(g)$$

12 $$2ZnO+Fe{S}_{2}+1/3C=2ZnS+1/3F{e}_{3}{O}_{4}+1/3C{O}_{2}(g)$$

13 $$2ZnO+Fe{S}_{2}+1/2C=2ZnS+FeO+1/2C{O}_{2}(g)$$

14 $$2ZnO+2FeS+2C=2ZnS+2Fe+2CO(g)$$

15 $$2ZnO+2FeS+C=2ZnS+2Fe+C{O}_{2}(g)$$

16 $$ZnO+C=Zn(g)+CO(g)$$

The ΔG for these seven reactions are also shown from 100 °C to 1200 °C in Fig. 1. The addition of carbon not only promotes the sulfidation of ZnO but also eliminates the generation of SO_2_.^36^ For iron phases, conversion to Fe_2_O_3_ (Eq. 11) and Fe_3_O_4_ (Eq. 12) has lower ΔG than conversion to FeS at temperature below 200 °C, above which however the ΔG for Eq. 10 becomes more negative than the others and hence ZnS, FeS, and CO_2_ are preferentially generated after ZnO roasted with FeS_2_. The ΔG for Eqs. 14 and 15 indicate that FeS can react with ZnO into ZnS plus Fe in the presence of carbon, which is beneficial for the sulfidation of ZnO when the amount of FeS_2_ is insufficient. Additionally, the reduction of ZnO to gaseous Zn becomes thermodynamically feasible at about 950 °C and then the ΔG becomes more negative with the increase in temperature, but it is difficult to occur in the temperature range investigated unless the amount of the sulfidizing agents including FeS_2_ and FeS are inadequate.

**Figure 2:**
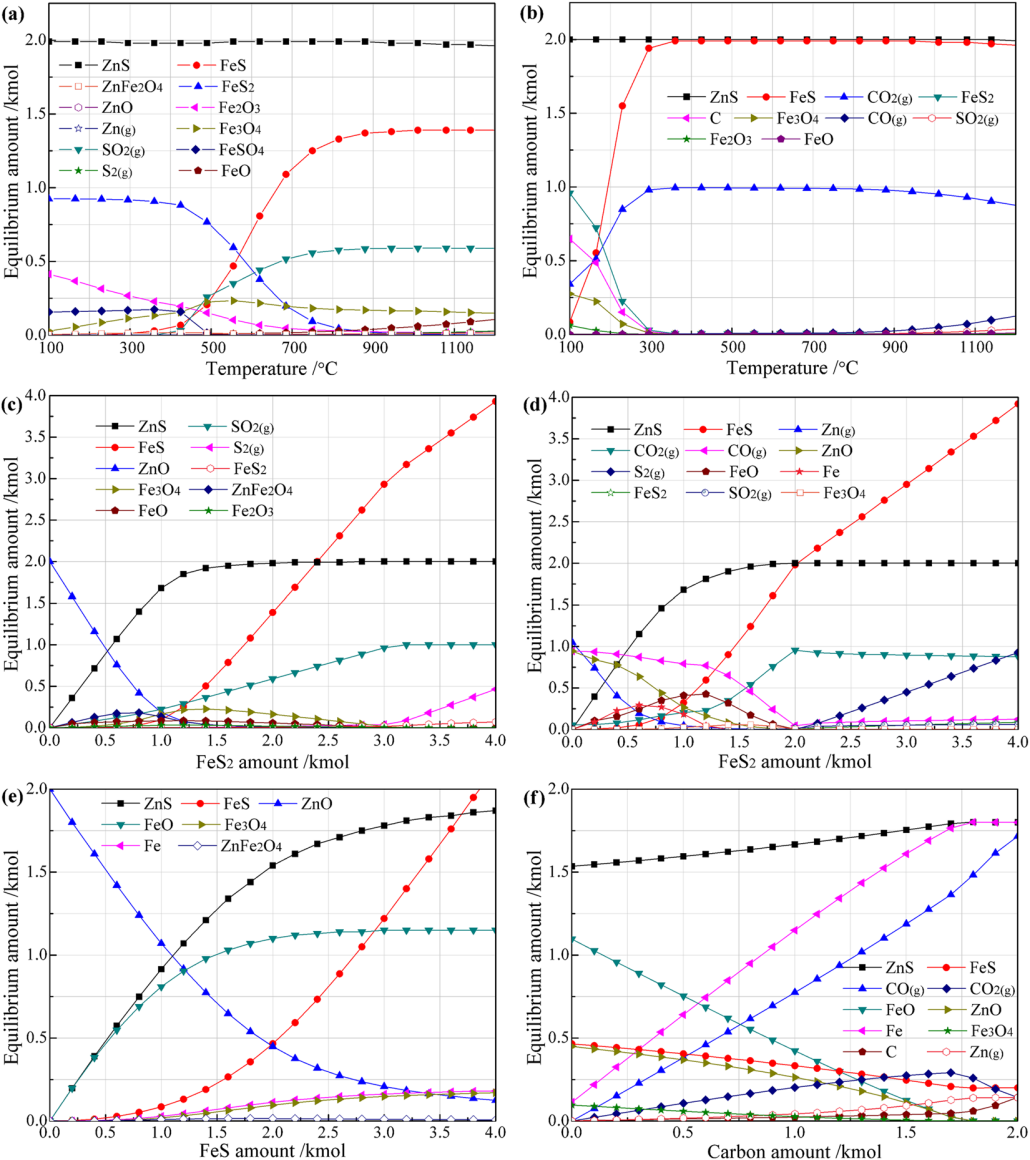
Equilibrium phase diagrams of ZnO (2 kmol) reacted with: (**a**) 2 kmol FeS_2_ at different temperatures, (**b**) 2 kmol FeS_2_ and 1 kmol carbon at different temperatures, (**c**) different FeS_2_ amount at 1000 °C, (**d**) different FeS_2_ amount and 1 kmol carbon at 1000 °C, (**e**) different FeS amount at 1000 °C, (**f**) 2 kmol FeS and different carbon amount at 1000 °C.

To fully understand the thermodynamic mechanism on the sulfidation of ZnO with pyrite, the equilibrium compositions of the reaction products were calculated making use of the Equilibrium Compositions module of Outokumpu HSC Chemistry 5.0^43^. The equilibrium composition was determined by the Gibbs free energy minimization method for isothermal, isobaric, and fixed mass conditions. For each calculation, the amount of ZnO was fixed at 2 kmol. Figure 2a indicates that the reaction products of ZnO and FeS_2_ are mainly composed of ZnS, Fe_2_O_3_, and Fe_3_O_4_ in the range of 100 °C to 400 °C, above which FeS begins to appear and its amount significantly increases with increasing temperature, while the amount of FeSO_4_ gradually decreases until it disappears at about 500 °C. When the temperature is above 700 °C, the reaction products are composed of mainly ZnS, FeS, SO_2_, and some Fe_3_O_4_. In the presence of carbon, the equilibrium compositions of the reaction products primarily include ZnS, Fe_3_O_4_, Fe_2_O_3_ as well as unreacted FeS_2_ and C at lower temperatures (Fig. 2b). With the increase in temperature, the amounts of FeS_2_, C, Fe_3_O_4_, and Fe_2_O_3_ gradually decrease as the amounts of FeS and CO_2_ increase. When the temperature is above 300 °C, the reaction products are mainly composed of ZnS, FeS, and CO_2_, indicating that the sulfidation reaction mainly follows Eq. 10.

Figure 2c and d show that the amount of FeS_2_ has a significant effect on the equilibrium compositions. The sulfidation extent of ZnO reaches its maximum when the dosage of FeS_2_ is 2 kmol, at which the reaction product consists of mainly ZnS, FeS, SO_2_, minor Fe_3_O_4_, and trace FeO for the case without carbon addition, while it mainly consists of ZnS, FeS, and CO_2_ for the case with the addition of 1 kmol carbon. When the dosage of FeS_2_ is insufficient, FeS is slight, or even none, due to the reaction of ZnO with the generated FeS. Figure 2d indicates that the reduction of ZnO can occur at low FeS_2_ amount, but the reaction can be effectively inhibited by increasing the amount of sulfidizing agent.

Our mentioned previously, FeS can act as the sulfidizing agent for the sulfidation of ZnO when the amount of FeS_2_ is insufficient. The effects of FeS amount and carbon amount on the equilibrium compositions of the reaction products were therefore investigated by thermodynamic calculation. Figure 2e reveals that the reaction product is mainly composed of ZnS, FeO, and unreacted ZnO or FeS, suggesting that the reaction primarily follows Eq. 9 in the absence of carbon. With increasing the amount of FeS, the amounts of ZnS and FeO gradually increase while the amount of ZnO decreases in the range investigated, but the change speeds gradually become slow and thus ZnO always exists in the reaction products. This indicates that FeS is relatively difficult to react with ZnO in comparison with FeS_2_. Figure 2f demonstrates that the addition of carbon can enhance the reaction of ZnO with FeS, but excess carbon will cause the reduction of ZnO to gaseous Zn and hence an appropriate amount of carbon is necessary for the sulfidation of ZnO.

**Figure 3:**
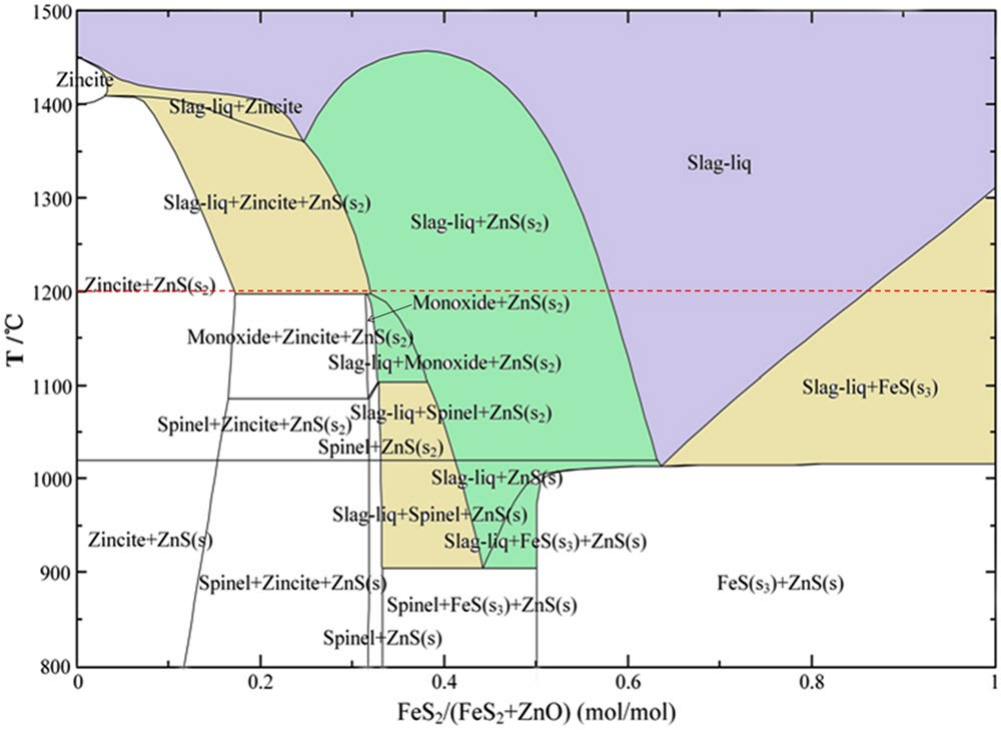
Phase diagram of FeS_2_-ZnO-CO system (PCO = 0.1 atm).

Our previous studies indicate that the formation of liquid phase during the roasting plays an important role in the growth of synthetic ZnS particles. To investigate the effects of reaction conditions on the formation of liquid phase and the stability areas of mineral phases, the phase diagram of FeS_2_-ZnO-CO system (Fig. 3) was calculated using the Phase Diagram module of FactSage 7.0. The partial pressure of CO was fixed at 0.1 atm. The stability areas containing liquid phase were marked with color and that without liquid phase are white. The composition of reactants has a significant influence on the formation of low melting compounds during heating process. When the mole ratio of FeS_2_/(FeS_2_ + ZnO) is in the range of 0.33 to 0.5, liquid phase begins to appear in the reaction system of ZnO with FeS_2_ at about 900 °C. When the mole ratio is without the range, higher temperature is required for the formation of liquid phase in the sulfidation system. Fortunately, the optimized dosage of FeS_2_ for the sulfidation of ZnO is in the range of 0.33 to 0.5, based on the above thermodynamic analysis and our previous experimental results, indicating that pyrite instead of sulfur as the sulfidizing agent is conducive to forming liquid phase and thus enhancing the growth of ZnS particles. It is also found that increasing temperature not only favors the formation of liquid phase but also enhances the sulfidation of zinc, because the stability area of spinel (ZnFe_2_O_4_) becomes smaller with the increase in temperature. As a conclusion, high temperature is helpful for both the sulfidation of ZnO and the growth of synthetic ZnS particles, but too high temperature (above 1200 °C) will cause the loss of zinc and the increase in energy consumption.

### Sulfidation behaviors

Although the reaction mechanism of ZnO with FeS_2_ has been revealed by the thermodynamic analysis, it is essential to detailedly investigate the sulfidation behavior of ZnO roasted with FeS_2_ by various experiments because the occurrence of a reaction also depends on the kinetic factors. A series of TG-DSC experiments were first performed to illustrate the sulfidation behavior of ZnO with pyrite in the absence and presence of carbon. Meanwhile, the decomposition behavior of pyrite was also investigated to help deduce the mechanism on the sulfidation of ZnO with pyrite.

**Figure 4:**
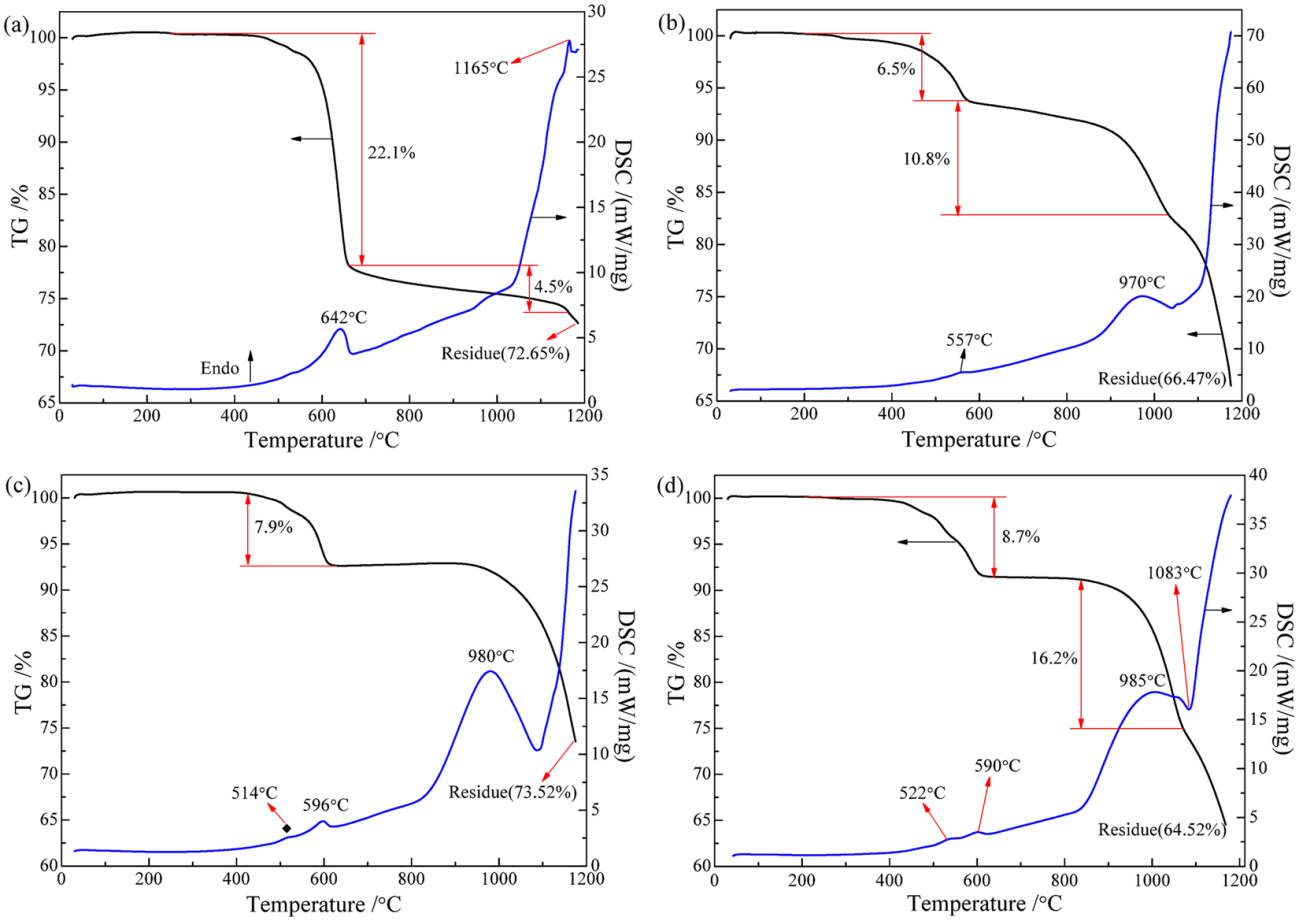
TG-DSC curves of non-isothermal process: (**a**) FeS_2_, (**b**) FeS_2_ + C, (**c**) ZnO + FeS_2_, (d) ZnO + FeS_2_ + C.

In Fig. 4a, three weight loss stages are observed in the TG curve of pyrite decomposition under nitrogen atmosphere. The first stage with mass loss of 22.1% approximately in the range of 410 °C to 665 °C is attributed to the release of gaseous sulfur produced by the decomposition of pyrite to pyrrhotite (Fe_1-*x*_S)^44^. The second one with mass loss of 4.5% from 665 °C to 1163 °C is ascribed to the decomposition of pyrrhotite to troilite (FeS). The total weight loss of the two stages is as high as 26.6%, suggesting that the decomposition of pyrite to pyrrhotite has been completed. However, with further increasing temperature, the TG curve still decreases significantly, which implies that the troilite generated can be decomposed into iron and gaseous sulfur at high temperatures (above 1160 °C). It is observed that the endothermic peak appeared at 642 °C is the most obvious in the DSC curve of pyrite decomposition. The TG-DSC curves of pyrite with 5% carbon (Fig. 4b) indicate that the addition of carbon has a significant effect on the decomposition behavior of pyrite. The TG curve can also be considered as consisting of three weight loss stages. The first one with mass loss of 6.5% from 225 °C to 577 °C is mainly attributed to the decomposition of pyrite to pyrrhotite, implying that the addition of carbon probably played an induction role in the decomposition and thus resulted in the decrease of initial reaction temperature. Correspondingly, an endothermic peak is observed at 557 °C, which is weaker than that at 642 °C in Fig. 4a. This indicates that, to some extent, the addition of carbon could slow down the reaction speed of pyrite to pyrrhotite, which is conducive to controlling the release speed of gaseous sulfur from pyrite and thus increasing the utilization rate of sulfur. The second with mass loss of 10.8% in the range of 577 °C to 1035 °C probably resulted from the reactions of pyrite to pyrrhotite and pyrrhotite to troilite. The endothermic peak clearly observed at about 970 °C confirms the decomposition of pyrrhotite. Above 1000 °C, the TG curve begins into the third mass loss stage, and with increasing temperature the mass loss decreases gradually, which is attributed to the reaction of pyrrhotite to troilite and then the decomposition of troilite to iron.

Figure 4c and d show the TG-DSC curves of the mixtures of zinc oxide and pyrite in the absence and presence of carbon, respectively. The dosage of pyrite is 0.6 mol per mole of ZnO and the dosage of carbon is 5 wt.% with respect to pyrite. In the absence of carbon, with increasing temperature the TG curve does not decrease until above 400 °C, implying that the sulfidation of ZnO began to occur after the decomposition of pyrite. This indicates that the sulfidation is mainly through the reaction of ZnO with the gaseous sulfur generated from the decomposition of pyrite. A mass loss of 7.9% observed in the TG curve from 410 °C to 630 °C is ascribed to the release of SO_2_ generated by the sulfidation of ZnO and to the release of gaseous sulfur by the decomposition of pyrite. Then, the TG curve almost stays at a constant value in the range of 630 °C to 930 °C, because the decomposition speed of pyrite was so slow that all the gaseous sulfur generated was converted into zinc sulfides. Above 930 °C, the TG curve gradually decreases with the increase in temperature and an endothermic peak is found at about 980 °C, which is attributed to the further decomposition of pyrite. With the addition of carbon, the TG-DSC curves are similar to that without carbon, but the initial temperature of weight loss is lower than that without carbon, because the initial decomposition temperature of pyrite was decreased by adding carbon. An exothermic peak is found at 1083 °C, indicating the occurrence of ZnO sulfidation. More weight loss confirms that the decomposition of pyrite and the occurrence of ZnO sulfidation could be enhanced by the addition of carbon.

**Figure 5:**
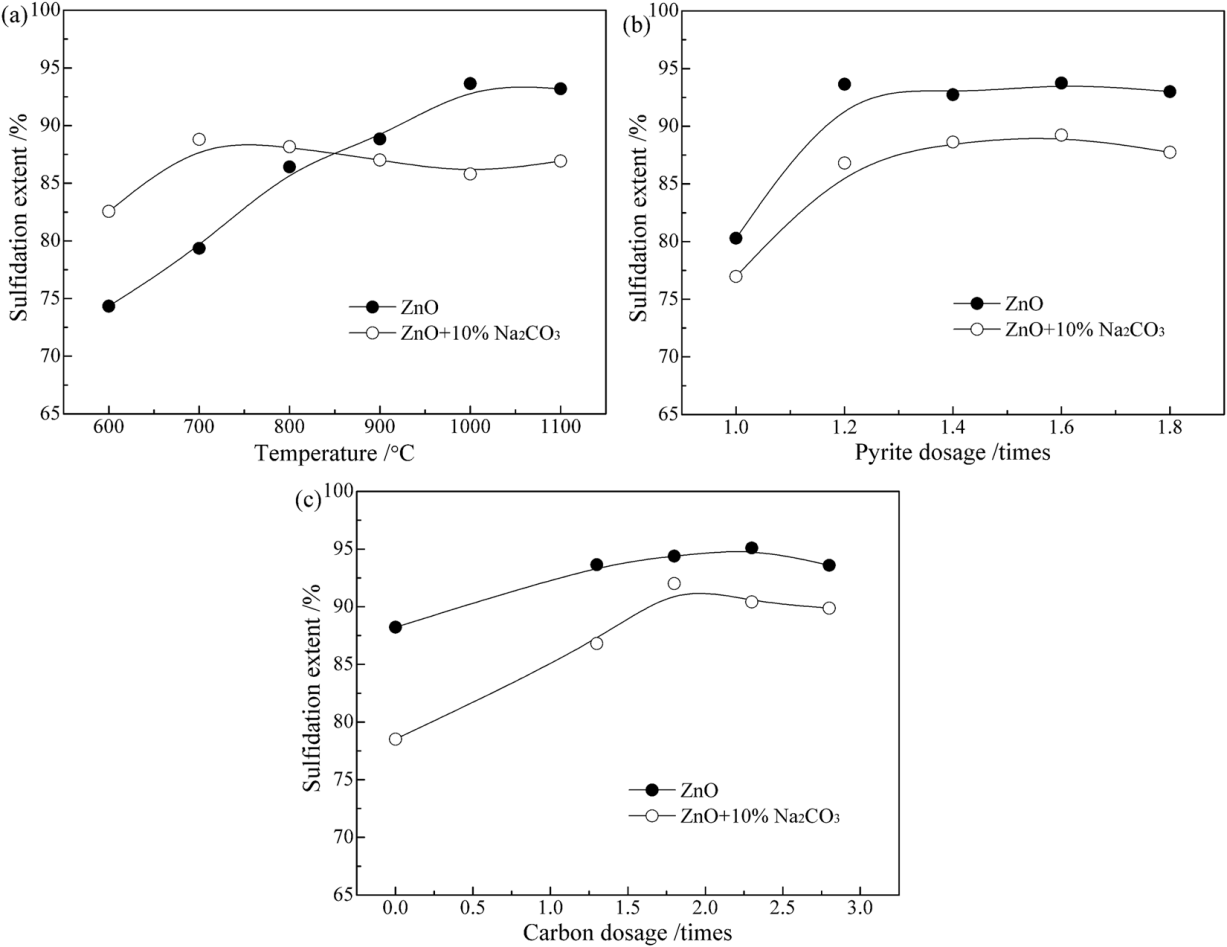
Effects of roasting conditions on the sulfidation behaviors of ZnO: (**a**) temperature, (**b**) pyrite dosage, (**c**) carbon dosage.

Based on the above studies, a series of the roasting experiments on a laboratory scale were carried out to investigate the effects of operating conditions on the sulfidation behaviors of ZnO. As shown in Fig. 5a, roasting temperature has a significant influence on the sulfidation of ZnO. With the increase in temperature, the sulfidation extent of zinc gradually increases until it reaches the maximum at 1000 °C, after which the value however decreases slightly. The addition of Na_2_CO_3_ has a positive effect on increasing the sulfidation extent of zinc at lower temperatures (below 850 °C), but a negative effect at higher temperatures. Figure 5b shows the effect of pyrite dosage on the sulfidation extent of zinc. The value significantly increases as the dosage of pyrite increases from 1.0 to 1.2 times of the theoretical value (the mole ratio of FeS_2_ to ZnO is 0.5). Thereafter, the value has no significant variation, indicating that the dosage of pyrite at 1.2 times was sufficient. Obviously, the addition of Na_2_CO_3_ has a negative influence on the sulfidation of ZnO in the pyrite dosage range investigated. Figure 5c demonstrates that the addition of carbon could promote the sulfidation of ZnO with pyrite, especially for with the addition of Na_2_CO_3_. However, too much carbon is bad for the sulfidation of ZnO as well as the recovery of ZnS, because the residual carbon can absorb flotation reagents in subsequent process. Therefore, 1.8 times of the theoretical value (the mole ratio of carbon to ZnO is 1/6) was chosen as the optimized dosage of carbon, under which the sulfidation extent of ZnO was as high as 94.38%.

### Phase transformations

**Figure 6:**
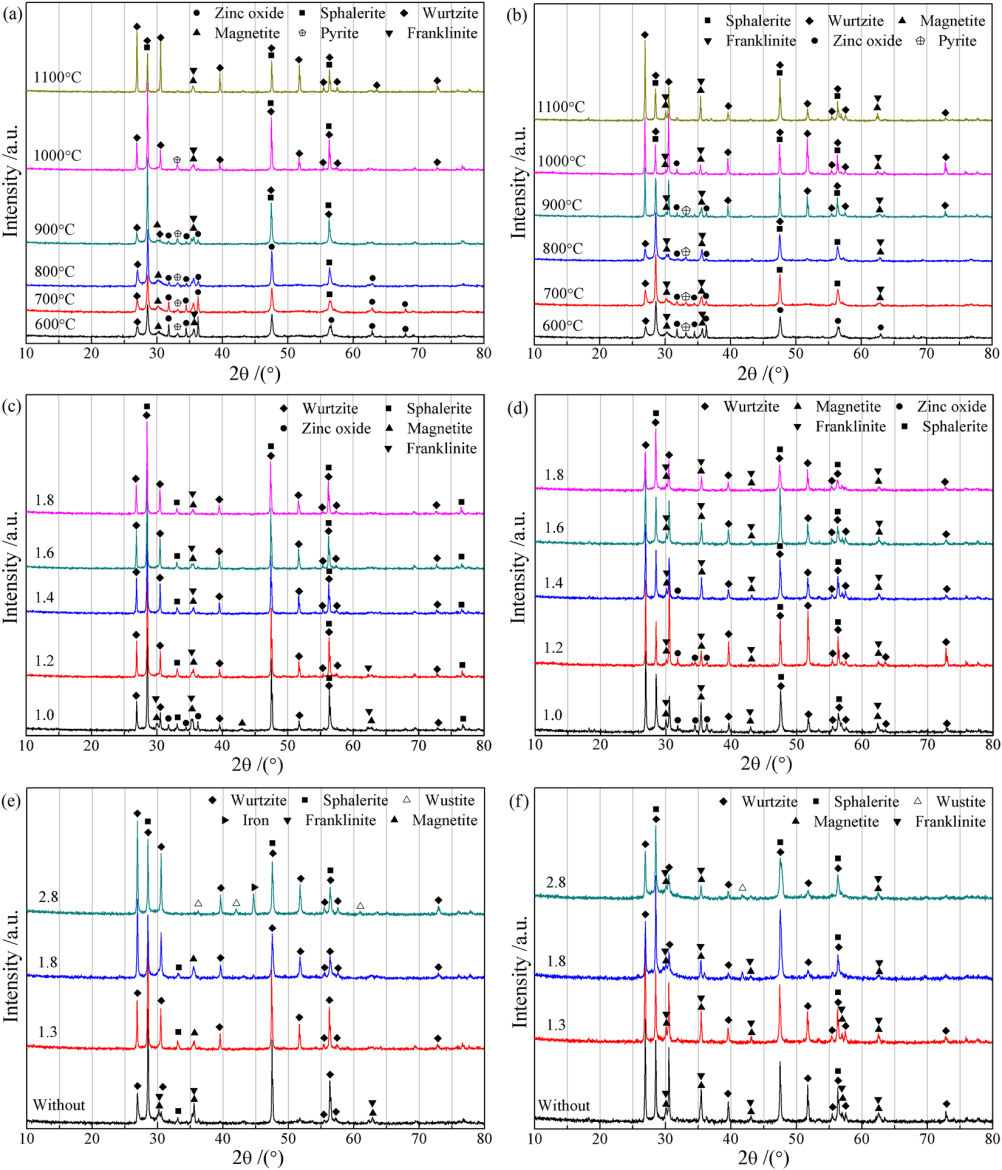
XRD patterns of the roasted ZnO: (**a**) at different temperatures, (**b**) at different temperatures with 10% Na_2_CO_3_, (**c**) with various pyrite dosages, (**d**) with various pyrite dosages and 10% Na_2_CO_3_, (**e**) with various carbon dosages, (**f**) with various carbon dosages and 10% Na_2_CO_3_.

To investigate the phase transformations during the sulfidation process, the samples roasted at different conditions were subjected to XRD analysis. As shown in Fig. 6a, with increasing temperature from 600 °C to 1000 °C, the diffraction peaks of ZnO and FeS_2_ decrease gradually, while the peaks of ZnS (sphalerite plus wurtzite) increase significantly, indicating that increasing temperature favors the sulfidation of ZnO. When the temperature is above 1000 °C, the sphalerite generated will convert into wurtzite, which is easy to sublimate into gas at high temperatures. This is the reason for the decrease of the sulfidation extent of zinc at 1100 °C in Fig. 5a. With the addition of Na_2_CO_3_ (Fig. 6b), the intensity of ZnO peaks is weaker than that without Na_2_CO_3_ at the range of 600 °C to 800 °C, which confirms the conclusion that Na_2_CO_3_ addition plays a positive role in the sulfidation of ZnO at lower temperatures. Meanwhile, the peaks of magnetite/franklinite are higher than that without the addition of Na_2_CO_3_, especially at higher temperatures, implying that the addition of Na_2_CO_3_ could increase the oxygen partial pressure of the sulfidation system. It is also found that the addition of Na_2_CO_3_ could enhance the conversion of sphalerite to wurtzite. For instance, the temperature required for the fast transformation of sphalerite to wurtzite is 1100 °C in the absence of Na_2_CO_3_, but it decreases to 900 °C in the presence of Na_2_CO_3_.

Figure 6c and d present the XRD patterns of the ZnO roasted with different pyrite dosages in the absence and presence of Na_2_CO_3_, respectively. Without the addition of Na_2_CO_3_, the roasting products are composed of mainly sphalerite, wurtzite, magnetite/franklinite, and minor zinc oxide according to the XRD analysis when the dosage of pyrite is the theoretical value, above which the peaks of zinc oxide disappear and the XRD patterns have no significant change as the dosage of pyrite increases from 1.2 to 1.8 times of the theoretical value. With the addition of Na_2_CO_3_, the phase compositions of the roasting products are similar to that without Na_2_CO_3_, but the diffraction peaks of ZnO have never disappeared until the dosage of pyrite is more than 1.4 times of the theoretical value, suggesting that the addition of Na_2_CO_3_ requires more dosage of pyrite for the sulfidation of ZnO.

Figure 6e and f show the effects of carbon dosage on the phase transformations during the roasting process in the absence and presence of Na_2_CO_3_, respectively. Obviously, the dosage of carbon has a significant influence on the phase compositions of the sulfidation products. Without the addition of Na_2_CO_3_, the diffraction peaks of wurtzite gradually increase, while the peaks of sphalerite and magnetite/franklinite decrease, as the dosage of carbon increases. When the dosage of carbon reaches up to 2.8 times of the theoretical value, the diffraction peaks of magnetite/franklinite have disappeared, while the peaks of wustite and iron appear in the XRD patterns.

### Microscopic morphology properties

**Figure 7:**
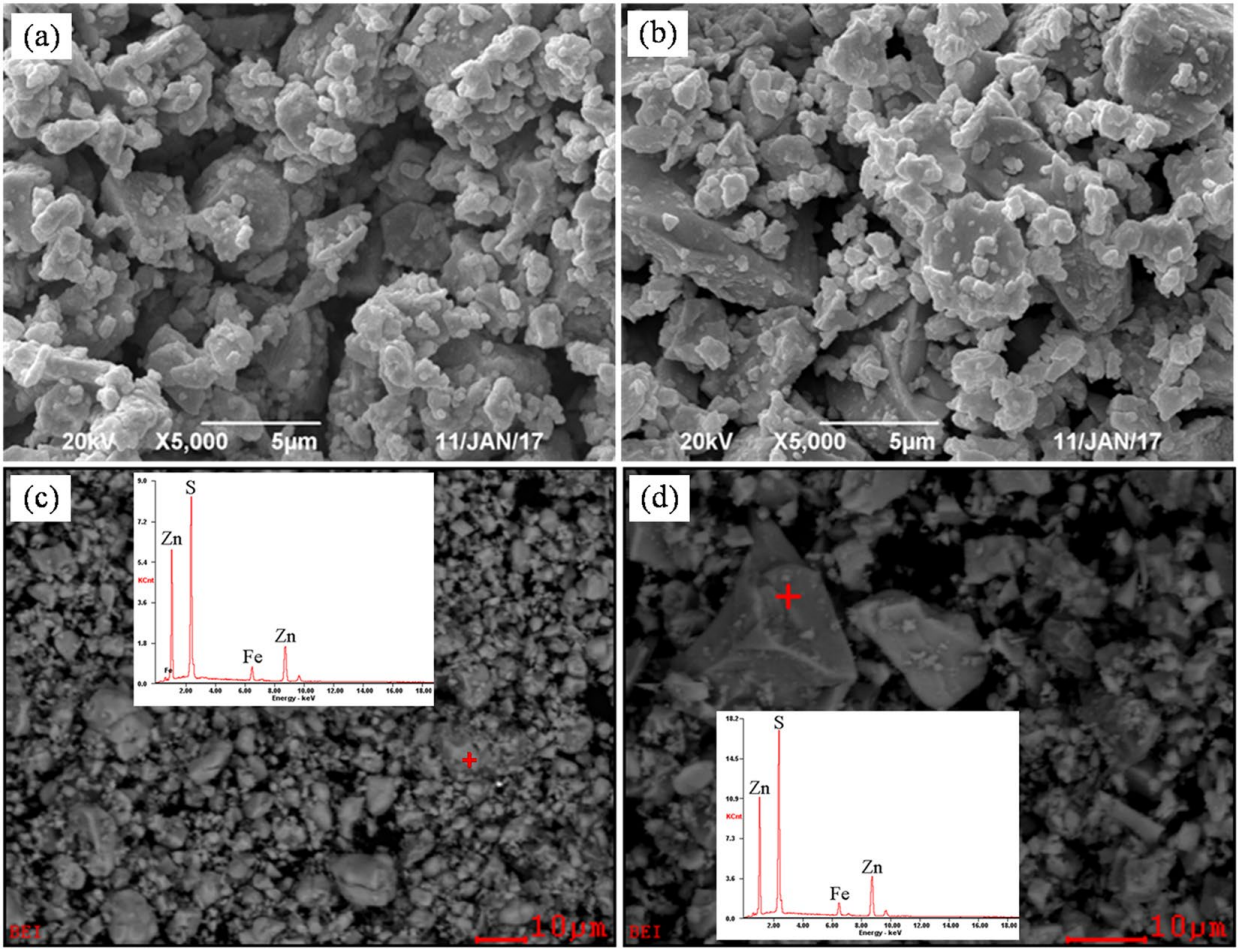
SEM-EDS results of the sulfidation roasted ZnO: (**a**) SEM image of the sample roasted without additive, (**a**) SEM image of the sample roasted with Na_2_CO_3_, (**c**) BSE-EDS images of the sample roasted without additive, (**d**) BSE-EDS images of the sample roasted with Na_2_CO_3_.

Based on the above discussion, the addition of Na_2_CO_3_ has no positive effect on improving the sulfidation extent of ZnO, but it is uncertain that whether or not the addition of Na_2_CO_3_ can play a positive role in the formation and growth of the ZnS crystals generated. The samples roasted without and with 10% Na_2_CO_3_ at 1000 °C were therefore investigated by SEM-EDS analysis (Fig. 7). The results indicate that the addition of Na_2_CO_3_ can enhance the formation and growth of the synthetic ZnS during the roasting process. The BSE images show the roasting product without Na_2_CO_3_ is fine, loose, and no clear boundary between different particles, but the sample roasted with Na_2_CO_3_ has a larger particle size, more compact structure, and clear edges and corners between different particles. This is attributed the fact that sodium carbonate is a low melting point compound (about 851 °C) and thus its addition promoted the formation of liquid phase, which could enhance the aggregation of fine ZnS grains by liquid bridge connections.

### Surface property analysis

**Figure 8:**
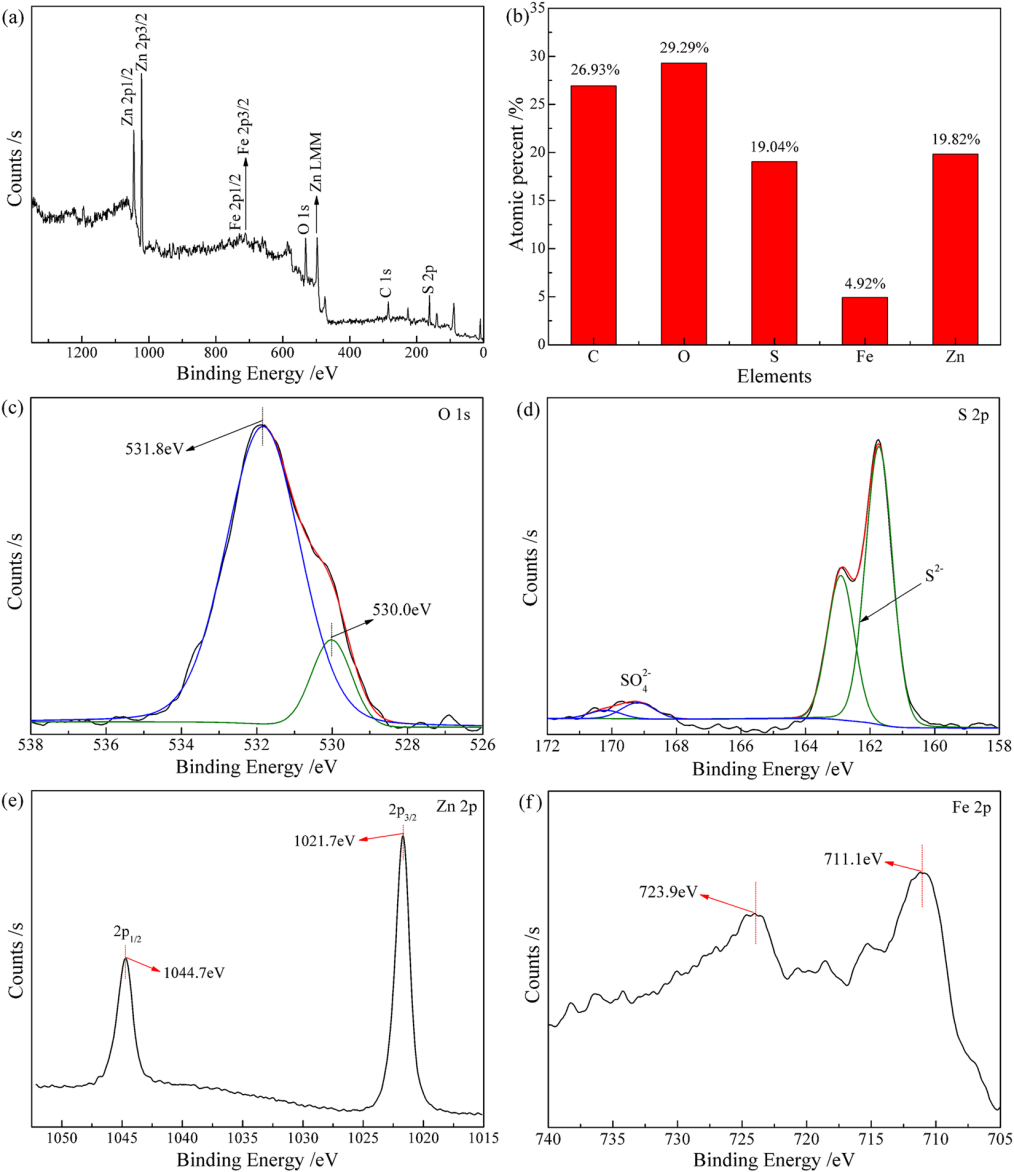
Surface properties of the ZnO roasted with pyrite: (**a**) XPS survey spectra, (**b**) elemental composition, and high-resolution scans for (**c**) O 1 s, (**d**) S 2p, (e) Zn 2p, and (**f**) Fe 2p electrons.

The surface elemental composition and chemical status of the ZnO roasted with pyrite in the presence of carbon at 1000 °C were investigated by XPS. A wide survey scan of XPS spectra was taken in the range of 0 to 1350 eV (Fig. 8a). The surface of the roasted ZnO contained Zn, Fe, S, O and C, but C was introduced onto the surface in XPS analysis. As shown in Fig. 8b, the atomic percentages of Zn, Fe, S, and O are 19.82%, 4.92%, 19.04%, and 29.29%, respectively. It is interesting that the content of O is relatively high. The O 1 s signal was deconvolved into two Gaussian fitted peaks with the binding energies of 531.8 eV and 530.0 eV (Fig. 8c), which respectively correspond to absorbed hydroxyl oxygen and surface lattice oxygen according to literatures^45–47^. The high-resolution spectrum of S 2p (Fig. 8d) reveals that the sulfur species on the surface mainly include sulfides and sulfates^48,49^. Combined Fig. 8b with Fig. 6, it can be deduced that the sulfides are mainly sphalerite and wurtzite and that the surface lattice oxygen probably derived from zinc oxide, iron oxides and zinc/iron sulfates, implying that the sulfidation of ZnO with pyrite belongs to surface chemical reaction and relates to the migration of oxygen from the inside of the sample particles to their surfaces^50,51^. In Fig. 8e, the binding energy of Zn 2p_3/2_ peak (1021.7 eV) is slightly lower than that of natural sphalerite (1021.8 eV)^39^, which is attributed to the presence of wurtzite. The shape and position of Fe 2p peak (Fig. 8f) demonstrate that the iron was in the form of Fe^3+^^52^, indicating that the iron on the surface of the resulting sample is easier to be oxidized.

## Conclusions

Thermodynamic and experimental studies indicate that ZnO can be converted into zinc sulfides by roasting with pyrite. The sulfidation of ZnO occurs mainly through reacting with the gaseous sulfur generated by the decomposition of pyrite, which proceeds through a multi-step process in the sequence of pyrite → pyrrhotite → troilite → iron. Pyrite instead of sulfur as the sulfidizing agent can not only relieve the volatilization loss of sulfur but also enhance the formation of liquid phase during the roasting process and thus facilitate the growth of ZnS particles. However, the speed of pyrite decomposition is larger than that of ZnO sulfidation and hence the roasting should be carried out under a closed condition to avoid the escaping of gaseous sulfur. The presence of carbon not only eliminates the release of SO_2_, but also decreases the decomposition temperature of pyrite and promotes the sulfidation of ZnO. The addition of Na_2_CO_3_ promotes the sulfidation of ZnO at lower temperatures (below 850 °C) and enhances the growth of ZnS particles but has a negative effect on the sulfidation at higher temperatures. XPS analysis reveals that the sulfidation reaction belongs to surface chemical reaction and relates to the migration of oxygen from the inside of ZnO to its surface. The thermodynamic calculation is in good agreement with the results obtained by experiments.
